# Supplementary material for: Altered expression of Notch1 in Alzheimer's disease
Source: PLoS One. 2019 Nov 26;14(11):e0224941. doi: 10.1371/journal.pone.0224941 (PMC6879159; doi:10.1371/journal.pone.0224941)
Supplement: S1 Fig — (PDF) [file pone.0224941.s001.pdf]

Supporting Information

Supplementary Figure 1. Original uncropped and unadjusted full blots of Fig 2b, 2c.

Boxes highlighted lanes used in Figure 2.

Figure 2b

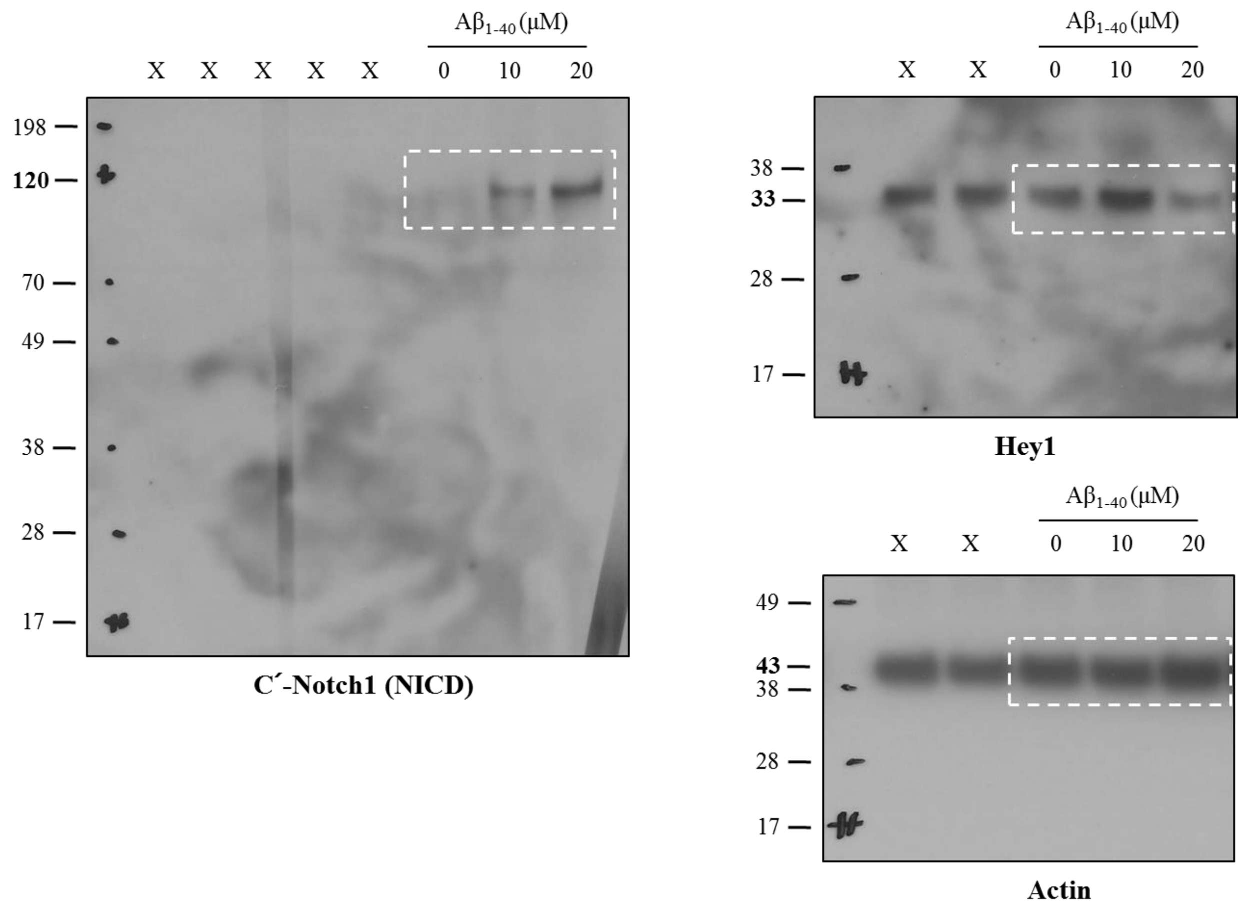

Figure 2c

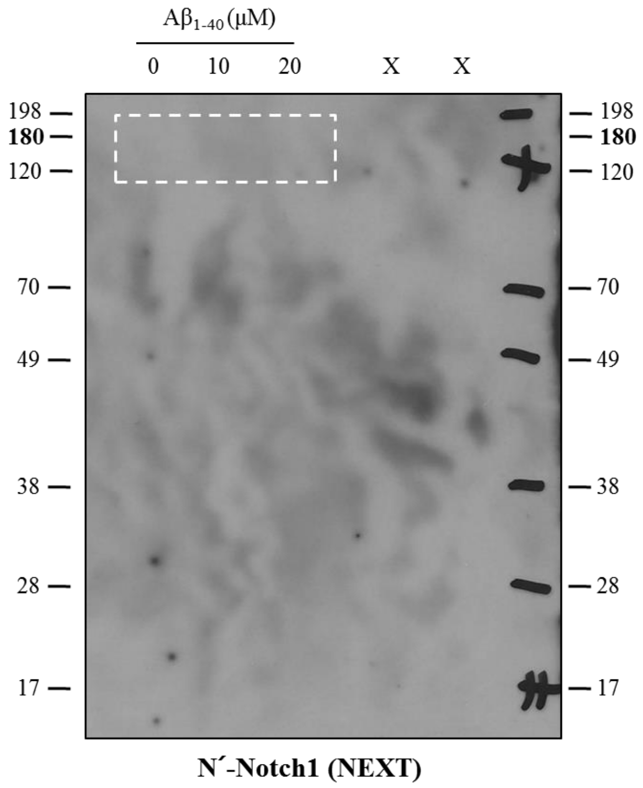
Aβ1-40 (μM)
0 10 20 X X
198
180
120
70
49
38
28
17
198
180
120
70
49
38
28
17
N´-Notch1 (NEXT)
