# Supplementary material for: Altered expression of Notch1 in Alzheimer's disease
Source: PLoS One. 2019 Nov 26;14(11):e0224941. doi: 10.1371/journal.pone.0224941 (PMC6879159; doi:10.1371/journal.pone.0224941)
Supplement: S2 Fig — (PDF) [file pone.0224941.s002.pdf]

Supplementary Figure 2. Original uncropped and unadjusted full blots of Fig. 3b, 3c. (PDF)

Boxes highlighted lanes used in Fig 3.

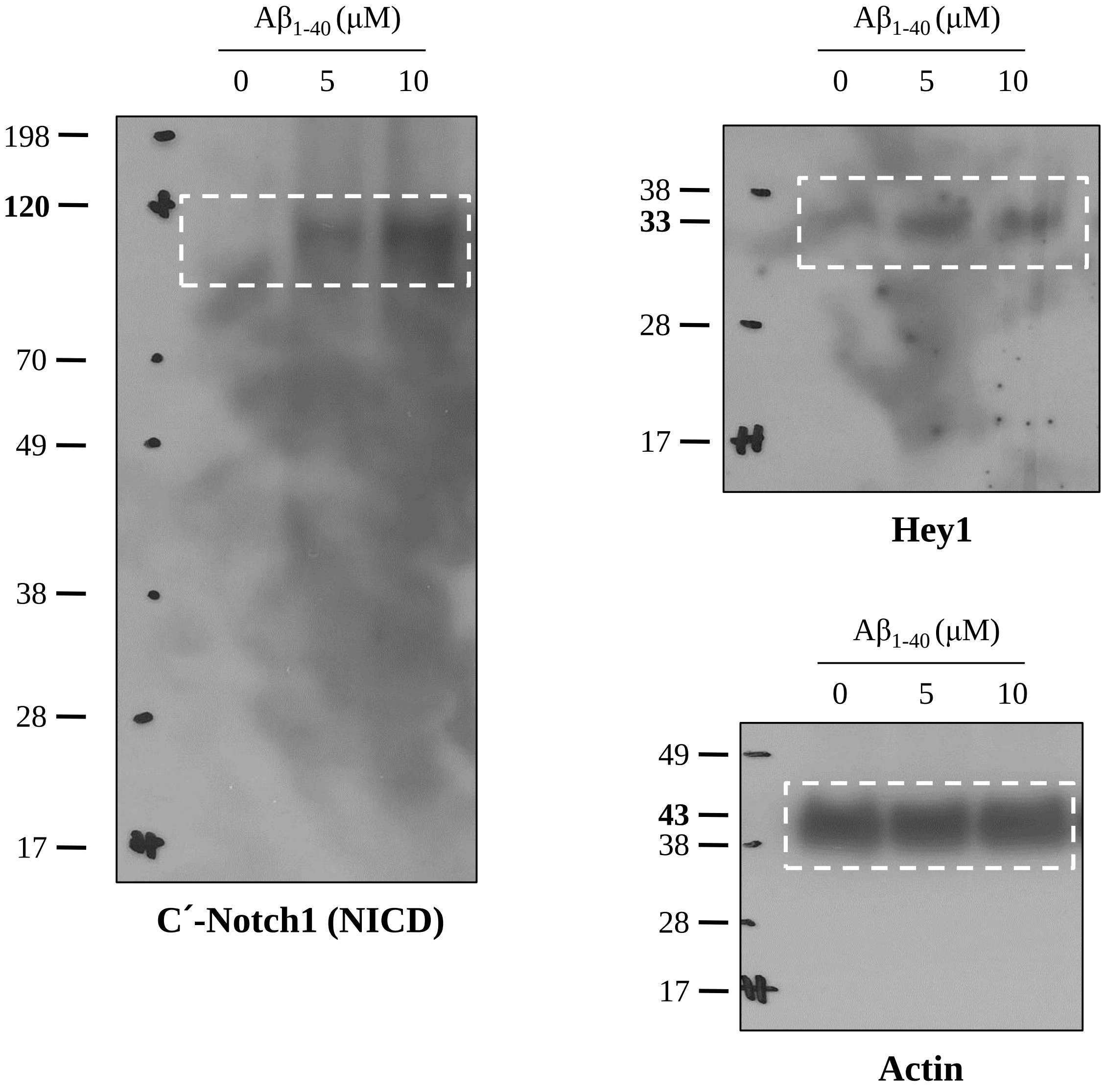

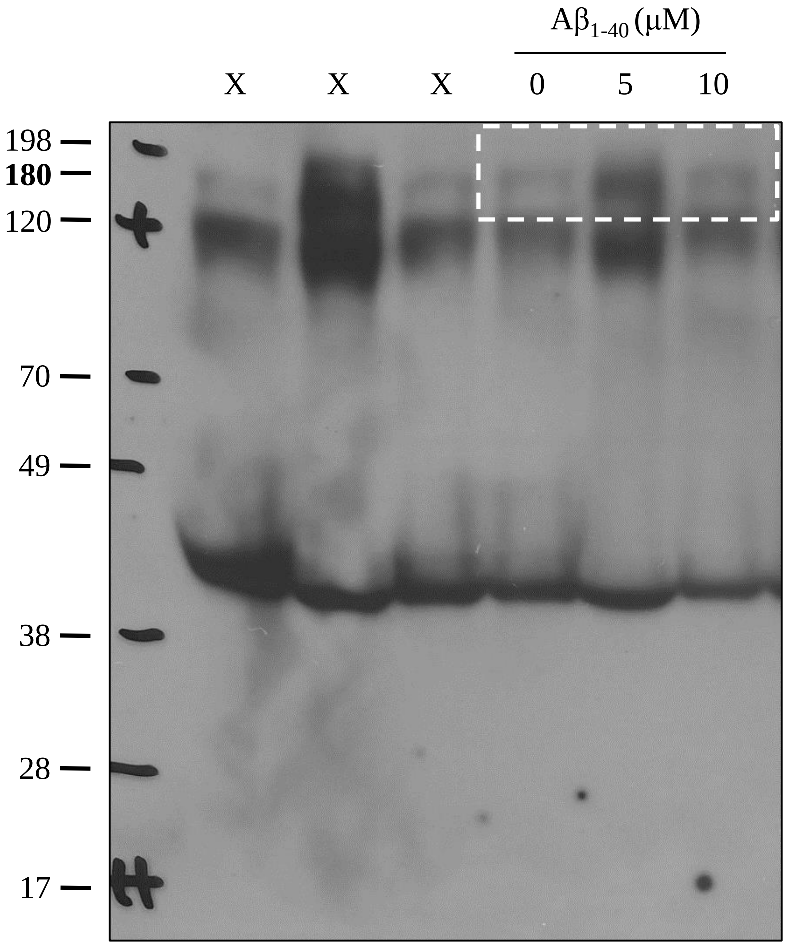

N´-Notch1 (NEXT)
