# Supplementary material for: Altered expression of Notch1 in Alzheimer's disease
Source: PLoS One. 2019 Nov 26;14(11):e0224941. doi: 10.1371/journal.pone.0224941 (PMC6879159; doi:10.1371/journal.pone.0224941)
Supplement: Attachment — Submitted filename: Response to Reviews_Revision_20190920.docx [file pone.0224941.s003.docx]

**Response to Reviews**

PLOS ONE

Manuscript number: **PONE-D-19-18002**

Title: Altered expression of Notch1 in Alzheimer′s disease

Corresponding Author: Dr. Young Ho Koh

Author: Sun-Jung Cho, Sang-Moon Yun, Chulman Jo, Jihyun Jeong, Moon Ho Park, Changsu Han and Young Ho Koh

**Reviewers' comments:**

**Reviewer #1:**

| **5 Major points** | | |
| --- | --- | --- |
| No. | *Reviewer’s comments* | *Our response* |
| 1 | • Table1: They also should demonstrate the specificity of the antibody. | As your suggestion, we added a new figure (**Fig 1a**) which shows the specificity of the Notch1 antibody used for ELISA.    **Fig 1. Comparison of the plasma levels of sNotch1.** (a) Standard curve of the human Notch1 ELISA kit. |
| 2 | • Table1: The bar plot would help to understand the variations of the data. | As your suggestion, we added a new figure (**Fig 1b**) which shows the camparision of plasma sNotch1 concentration between control, MCI, and AD by bar plot.    **Fig 1. Comparison of the plasma levels of sNotch1.** (b) Plasma sNotch1 concentration was measured by ELISA. The differences in the relative amounts of sNotch1 were compared between control, MCI, and AD by means of Mann-Whitney’s U-test within different groups. |
| 3 | • Figure 3: The authors described that mRNA levels of Notch1 are increased iPSC-derived AD neurons. Do you think it is Notch1 specific? The authors also should measure at least one more gene in these samples, hey-1 or hes-5. | As your suggestion, we added a new figure (**Fig 5b and c**). We also measured the *Hey1* and *Hes5* mRNA levels in iPSC-derived AD neurons and healthy neurons in **Figure 5**.    **Fig 5. *Notch1* mRNA expression in human iPSC-derived neuronal cells.** (a) Relative Notch1 mRNA expression levels were measured in human iPSC-derived neuronal cells from an AD patient and a healthy control subject (n = 3). Human iPSC cells were differentiated to neurons by treating with neuronal differentiation media. *Notch1* mRNA expression was significantly increased in human iPSC-derived neuronal cells from an AD patient (* p < 0.05). (b-c) Relative *Hey1* and *Hes5* mRNA expression levels were measured (n = 3, respectively) and significantly increased in human iPSC-derived neuronal cells from an AD patient (both ** p < 0.01). |
| 4 | • Before resubmitting, I would recommend having the manuscript reviewed by a native English speaker. | We have checked the manuscript reviewed by a professional English editing service company. |
| 5 | • The manuscript should format according to the journal instruction. Some parts including reference part is not well formatted. | We corrected the error according to the journal instruction in the manuscript. |

**Reviewer #2:**

In this paper, the authors show that Notch1 signaling is modulated by pathological condition such as Alzheimer’s disease. The manuscript is well-written, but the current data is missing some points.

| **3 Major points** | | |
| --- | --- | --- |
| No. | *Reviewer’s comments* | *Our response* |
| 1 | • As mentioned by the authors, it is well known that Notch1 extra-cellular domain is cleaved by metalloprotease ADAM10. To understand how sNotch1 is increased in culture media need to confirm the ADAM 10 expression. | As your suggestion, we added a new figure (**Fig 4a and b**). We also measured the *ADAM10* mRNA levels in Aβ-treated ECs in **Figure 4**.    **Fig 4. ADAM10 mRNA expression in ECs by amyloid beta (Aβ).** HUVECs (a) and HBMECs (b) were treated with 10 μM Aβ_1-40_ peptides for 8 h or 24 h. Real-time PCR results showing relative mRNA expression levels of ADAM10 (n = 3). Treatment with 10 μM Aβ_1-40_ peptides significantly increased the ADAM10 mRNA expression levels (* *p* < 0.05 and ** *p* < 0.001, respectively).  Increased ADMA10 might affect to the cleavage of Notch1 extracellular domain under the Aβ-treated condition in ECs. So, it could be explain that secreted sNotch 1 extracellular domain (NEXT) is increased in HBMECs culture media. |
| 2 | • Since Notch1 ligands, Dll4 and Jag1, have opposite effects and is required to activate Notch1 signaling in endothelial cell differentiation in artery. Investigation of these protein will give more information about activation of Notch1. | Thank you for your comments.  We agree with the reviewer’s suggestion.  We mentioned at **Discussion and References** section as follow;  In **Discussion** section :  Notch signaling has a critical roles in arterial formation and maturation. Dll4 and Jagged1 are important Notch1 ligands and were shown to have opposing effects in developmental angiogenesis. But the role of Jagged1/Notch signaling remains incompletely understood[43]. Notch signaling is associated with several pathological conditions and recent study shows that Notch signaling foster macrophage maturation during ischemia causing inflammatory responses[44]. Further studies will be required to examine Jagged1 and Dll4 to understand the role of Notch signaling in AD.  In **References** section :  **Reference #43.** A.R. Pedrosa *et al.* Endothelial Jagged1 antagonizes Dll4 regulation of endothelial branching and promotes vascular maturation downstream of Dll4/Notch1. Arterioscler Thromn Vasc Biol. 2015; 35:1134-46.  **Reference #44.** K. Krishnasamy *et al.* Blood vessel control of macrophage maturation promotes arteriogenesis in ischemia. Nature comm. 2017; 8: 952. |
| 3 | • Notch1 signaling is activated by not only development stage but also pathological condition such as ischemic stroke, neuronal death, and followed inflammatory responses. Please, cite and discuss those related previous findings which will be helpful to understand role of Notch1 signaling in neurodegenerative diseases. | Thank you for your comments.  As your suggestion, we mentioned at **Discussion and References** section as follow;  In **Discussion** section :  Activation of Notch1 signaling induced by cerebral ischemia results in a protracted nuclear factor-κB (NF-κB)-driven microglia-mediated neuroinflammation and worsens ischemic brain damage and functional outcome[39]. Activated microglial cells are detected in peri-infarct areas in clinical stroke and though to orchestrate neuronal damages in the penumbra[10]. Recent studies raise the possibility of the links between the Notch signaling pathway and diverse pathological disorders including adaptive and innate immune responses, multiple sclerosis, inflammatory demyelinating disease, which is providing Notch as a novel prospective target for the treatment of neuroinflammation-related degenerative disorders.  In **References** section :  **Reference #10.** L. G. The possible contribution of microglia and macrophages to delayed neuronal death after ischemia. . Journal of Neurological science. 1993;114:119-22.  **Reference #39.** Zelan Wei SC, Thiruma V. Arumugam, Dong-Gyu Jo, He Li, and Sic L. Chan. Notch activation enhances the microglia-mediated inflammatory response associated with focal cerebral ischemia. Stroke 2011;42:2589-94. |
